# Higher expression of pseudouridine synthase 7 promotes non-small cell lung cancer progression and suggests a poor prognosis

**DOI:** 10.1186/s13019-023-02332-z

**Published:** 2023-07-07

**Authors:** Guihong Zhang, Yongde Zhu, Yonghuang Tan, Biao Chen, Shichao Shan, Gengyu Zhang, Jianjun Lu

**Affiliations:** 1 Department of Thoracic Surgery, The First Affiliated Hospital, Sun Yat-sen University, Zhongshan 2nd street, No. 58, Guangzhou, Guangdong 510080 China; 2 Emergency Department, Hainan Province Nongken Sanya Hospital, Jiefang 4th Road, No. 1154, Sanya, Hainan 571159 China; 3 Department of Thoracic Surgery, Cancer Center, Sun Yat-sen University, Dongfeng East Road, No. 651, Guangzhou, Guangdong 510060 China; 4 First School of Clinical Medicine, Guangdong Medical University, Wenming East Road, No. 2, Zhanjiang, Guangdong 524023 China

**Keywords:** Non-small cell lung cancer, Pseudouridine synthase 7, Prognosis, Survival

## Abstract

**Background:**

Lung cancer is currently the second most common cancer, and non-small cell lung cancer accounts for about 85% of cases. NSCLC has not been studied for pseudouridine synthase 7 (PUS), a member of the PUS family that is associated with cancer development. Here, we focused on the role and clinical significance of PUS7 in non-small cell lung cancer.

**Aim:**

To explore the role of PUS7 in NSCLC and its clinical significance.

**Methods:**

We downloaded datasets from the TCGA database and CPTAC database. In normal bronchial epithelial cells as well as NSCLC cell lines, RT-PCR and Western blot were used to quantify PUS7 expression. The role of PUS7 in NSCLC has been investigated by CCK8, migration assay, migration assay, and flow cytometry. PUS7 expression in tumor tissues was detected by immunohistochemical staining, and we evaluated the influence of PUS7 expression on the prognosis of NSCLC patients after surgery using Cox regression analysis, both univariate and multivariate.

**Results:**

NSCLC cell lines and tissues expressed high levels of PUS7, and PUS7 was found to influence the proliferation, migration, and invasion of cancer cells without affecting their apoptosis. There was a worse prognosis for NSCLC patients who have higher PUS7 expression, suggesting that PUS7 was an independent indicator of prognosis (*P* = .05).

**Supplementary Information:**

The online version contains supplementary material available at 10.1186/s13019-023-02332-z.

## Introduction

As of 2020, lung cancer is the second most common malignancy, accounting for 11.4% of the total new cases of malignant tumors, and it is estimated that about 800,000 people die of lung cancer (18%) globally every year [1]. Although the research and application of targeted drugs have progressed rapidly in the past 20 years, not all lung cancer patients can benefit from effective targeted therapy [2]. Inhibitors based on EGFR, ALK, ROS1, and other targets are not suitable for all NSCLC patients, and even if patients have targeted therapy based on specific targets, the results cannot avoid the outcome of drug resistance and progression [3]. Therefore, it is crucial to discover potential therapeutic targets for NSCLC to improve the overall prognosis of patients.

It is reported that the disorder of RNA pseudouridine (Ψ) modification is closely associated with the occurrence of cancer [4]. At least 10 Ψ synthases, covering five families, TruA, TruB, TruD, RluA, and Pus10, found in human cells, indicate specific localization and RNA targets in cells [5–7]. Previous studies have shown that at least four enzymes are involved in cancer and have shown the potential to influence the characteristic of cancer cells. For instance, the SRA1 mediated by PUS1 can bind to retinoic acid receptor-γ (RARγ) in melanoma cells and estrogen receptors in breast cancer cell lines [8]. Knockdown of PUS10 increases apoptosis mediated by TNF-related apoptosis-inducing ligand (TRAIL) in p53-negative cancer cells, although this effect may be triggered by the catalytic activity of pseudouridine [9]. Besides, PUS10 was remarkably up-regulated in lung cancer tissues compared to adjacent tissues. Although its pathogenic role in lung cancer is not clear, it is also suggested that it can be regarded as a related risk site [10]. Hepatocellular carcinoma [11], breast cancer [12], prostate cancer [13], colorectal cancer [14], and lung cancer [15] express DKC1 at high levels, and its expression is related to disease progression and prognosis.

Studies have shown that PUS7 can be found to mediate pseudouridylation at more than 200 sites after induction of heat shock in yeast, while downregulation of PUS7 reduces mRNA Ψ levels, suggesting a role in enhancing transcriptional stability [16]. Case reports and studies have shown that PUS7 mutations can cause human syndromes including intellectual disability, microcephaly, delayed speech, and aggressive behavior, and it has been confirmed that this disease is related to the reduction of Ψ levels of tRNA and mRNA caused by PUS7 mutations, suggesting the importance of RNA pseudouridylation in nervous system development [16–20]. Recently, it has been reported that PUS7 can serve as an underlying biomarker for glioma in a neurological tumor, glioblastoma [21], and up-regulated PUS7 increased Ψ modification in tRNA and decreased TYK2 translation can promote tumor progression through the interferon-STAT1 pathway. Studies have also found that high expression of PUS7 in GBM patients predicts a poor prognosis [22]. In addition, the significance of PUS7 has also been observed in other tumor studies. For example, the bioinformatic data analysis found that PUS7 can be considered a potential biomolecule for ovarian cancer [23]. Furthermore, it is reported that PUS7 can regulate the metastatic ability of colon cancer cells through the HSP90/PUS7/LASP1 axis [24].

## Materials and methods

### Bioinformatic data

To investigate PUS7 mRNA in human NSCLC tissue, some clinical data were obtained from The Cancer Genome Atlas (TCGA) (https://cancergenome.nih.gov) and The National Cancer Institute’s Clinical Proteomic Tumor Analysis Consortium (CPTAC) (https://proteomics.cancer.gov/).

### Cell lines and cell culture

Four human NSCLC cell lines (H1299, A549, H226, and H460) and normal bronchial epithelial cells (BEAS-2B) were gained from the American Type Culture Collection (ATCC, Manassas, VA, USA). The cells were inoculated into RPMI-1640 medium, DMEM medium (Gibco, Waltham, MA, USA), or modified F-12 K medium (Procell, Hubei, China) added with 10% fetal bovine serum, 100 U/mL penicillin, and 100 µg/mL streptomycin(Gibco, Waltham, MA, USA). All cells were grown in a 37 °C, humidified, 5% CO2 environment.

### Pus7 interference and overexpression

The interfering RNA (siRNA- PUS7) was purchased from Genepharma Corporation (Jiangsu, China), and its sequence was 5’-GCUAGGGAAUUUCAGCUAUTT-3’. Genepharma was employed to design a coding sequence for the human PUS7 overexpression plasmid, which was constructed into the pcDNA3.1 vector. According to the manufacturer’s protocol of Lipofectamine 3000 (Glpbio, Montclair, CA, USA), cells were transfected with siRNA or plasmids when inoculated in six-well plates for 8–12 h, and PUS7 expression was measured by western blot 48 h after cells were transfected.

### Protein extraction and western blot

RIPA cell lysate (Beyotime, Shanghai, China) was employed to collect proteins, which were segregated by 10% SDS-PAGE electrophoresis before electrotransferred to PVDF membranes (Millipore, Billerica, MA, USA). Next, 5% whole milk in TBST was employed to block nonspecific binding for 1 h. Protein bands were visualized with ECL luminescence solution on a Bio-Rad ChemiDoc XRS + Imaging System and then quantified with Image J. Antibodies and concentrations used in this study: anti-PUS7 (ab224119, Abcam, Cambridge, UK, 1:4000-1:1000), anti-GAPDH (60004-1, Proteintech, Hubei, China, 1:5000), anti-β-Actin (20536-1-AP, Proteintech, Hubei, China, 1:2500), goat anti-rabbit (SA00001-2, Proteintech, Hubei, China, 1:5000), horse anti-mouse (7076 S, CST, Massachusetts, USA, 1:2000).

According to the specification, we used a total RNA extraction kit (Yishan, Shanghai, China) to insulate and gain total RNA from cells, and then used a reverse transcription kit (Accurate, Hunan, China) to synthesize cDNA. Thereafter, qPCR was conducted on the LightCycle480 II system (Roche, USA), following the procedure given by the SYBR Green PCR kit (Accurate, Hunan, China). Reactions were performed in triplicate. Primer sequence: PUS7, sense: 5’- GGTGTGTCGCTGAAACGTG − 3’,

Antisense: 5’- AGTCATTCTGTAGCCCATCTTGA − 3’;

GAPDH, sense: 5’- CTGGGCTACACTGAGCACC − 3’,

Antisense: 5’- AAGTGGTCGTTGAGGGCAATG − 3’.

### CCK-8 assay

The cells were trypsinized, centrifuged, resuspended, counted, and added into 96-well plates with about 2000 cells per well. Six wells were repeated for each observation period in each group. Using the Cell Counting Kit-8 (Glpbio, Montclair, CA, USA), 100ul of 10% CCK8 working solution, diluted with serum-free medium, was added to each well. Then, the 96-well plates were put into a cell incubator for two hours. Then, using a Sunrise microplate reader (Tecan, Austria), each well was detected at 450 nm.

### Migration assay and invasion assay

8-µm-well chamber inserts for 24-well plates are used to test the migratory and invasive abilities of tumor cells. In the invasion assay, Matrigel (Corning, MA, USA) was diluted on ice and added to a chamber insert. After coagulation, about 60,000–80,000 cells resuspended in serum-free medium were inoculated into Falcon culture chambers, and a medium containing 20% FBS was used as an inducer outside the chamber inserts. In the cell migration assay, however, a culture chamber uncoated with Matrigel was used. Cells were cultured at 37 °C for 18–36 h and then fixed with 4% paraformaldehyde. A microscope (Leica DMI4000B, Munich, Germany) was used to count the number of cells invading and migrating in 5 different fields of view after staining the chamber inserts with 0.1-0.2% crystal violet. All groups were performed in triplicate. The counted cells were used to measure the migratory and invasive abilities of the cells.

### Flow cytometry

Apoptosis detection was conducted on a flow cytometry according to the instructions of Annexin V-FITC Apoptosis Detection Kit (KeyGEN, Jiangsu, China). After the transfected cells to be tested were digested with EDTA-free trypsin and washed thoroughly, 500ul of Binding Buffer was employed to resuspend the cells, followed by 5ul of Annexin V-FITC and 5ul of Propidium Iodide. The mixed liquid was transferred to a flow tube, followed by a reaction at room temperature for 5–15 min, and then detected on a flow cytometer (Beckman Coulter, California, USA). The above steps are all carried out in the dark.

### NSCLC patient data and pathological specimens

A total of 181 NSCLC patients who underwent surgery in the First Affiliated Hospital, Sun Yat-Sen University from April 2015 to September 2016 were included.

Patients were staged based on the 7th edition of the UICC TNM staging system for NSCLC.

Inclusion criteria: 1) NSCLC patients with stage IB-IIIA;

2) Patients underwent standard single lobectomy and hilar and mediastinal lymph node dissection, which was confirmed to be R0 resection according to postoperative pathological examination;

3) There are no contraindications for postoperative chemotherapy.

Exclusion criteria: 1) Patients with incomplete or missing clinicopathological data;

2) Patients with other uncured malignancies;

3) Patients with multiple primary lung cancers during the same period.

The study was approved by the Ethics Committee of the First Affiliated Hospital, Sun Yat-sen University.

### Immunohistochemical staining and scoring criteria

The tissue microarray (TMA) slides were dewaxed using xylene, followed by hydration in graded ethanol. According to the instructions of the immunohistochemical kit (Dako, Denmark), the slices were put into the immunohistochemical pretreatment system PT Link (Dako, Denmark) that had been preheated to 65 °C in advance, and the temperature was raised to 97 °C for 20 min. After that, it was automatically cooled to 70 °C, soaked in EnVision FLEX washing buffer for 5 min, and cooled to room temperature. EnVision FLEX Peroxidase Blocker was added dropwise to sections in a humidified chamber, followed by incubation at room temperature for 10–15 min. Then the slides were put in a moist box and incubated with an anti-PUS7 primary antibody working solution (ab224119, Abcam, Cambridge, UK, 1:400) for 1 h. Next, they were added with EnVision FLEX MOUSE (LINKER) dropwise and reacted at 37 °C for 15 min. EnVision FLEX HRP was added dropwise in the dark and reacted at 37 °C for 30 min and EnVision FLEX SUBSTRATE BUFFER was dropped onto the slices with 10-minute incubation. The slides were washed with clean water, then stained with hematoxylin, dehydrated, dripped with resin, and covered with coverslips. Finally, a digital pathology slide scanner was used to scan the slides (KF-TB-400, Jiangfeng, Jiangsu, China).

Tumor cells mainly express PUS7 proteins in the nuclei. A brown change in tissue on the slide signified a positive reaction. The positive intensity was measured, based on the following scoring criteria: 0 (negative), 1 (weak), 2 (moderate), or 3 (strong), and the proportion of positive cells was measured according to the following criteria: 0 (negative), 1 (1–25%), 2 (26–50%), 3 (51–75%), or 4 (76–100%). The result of multiplying the intensity of positive cells by the proportion of positive cells is the IHC score (0–12). All slides were scored by two independent pathologists with no prior knowledge of the patients’ clinical information beforehand, and they ultimately considered differences in scoring and reached an agreement.

### Quantification and statistical analysis

Analyses and tabulations were done with SPSS software (version 20.0) and Prism version 8.0. The chi-square test was employed to evaluate the differences in clinical data between the two groups, and the optimal cutoff value of the immunohistochemical scoring index was selected with the X-tile program [25]. Kaplan-Meier survival analysis was used to calculate and plot survival curves. To identify independent factors affecting survival, we performed univariate and multivariate Cox regression analyses. *P* < .05 indicates a statistical difference.

## Results

### NSCLC has a higher PUS7 expression

**Fig. 1:**
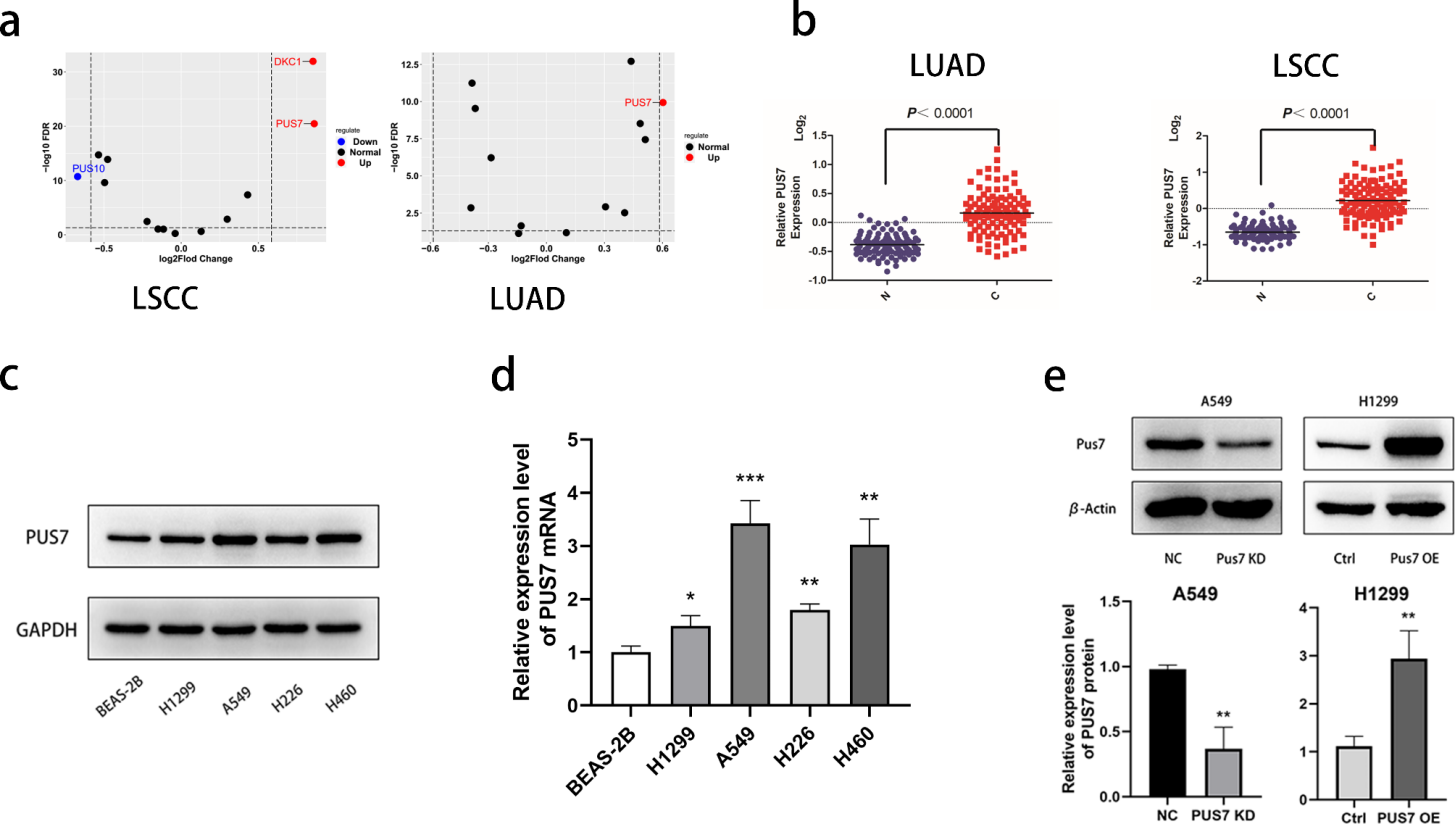
PUS7 is highly expressed in non-small cell lung cancer. **(a)** Expression of pseudouridine synthase family in NSCLC in TCGA. **(b)** Comparison of PUS7 expression in normal tissues and NSCLC at the mRNA level in CPTAC. **(c)** Western blot was used to determine the expression of PUS7 protein in NSCLC cell lines. **(d)** RT-PCR was used to determine the expression of PUS7 mRNA in NSCLC cell lines. **(e)** Western blot was used to determine the expression of PUS7 in the transfected tumor cells. All the data are shown as the mean ± s.d. *P < .05, **P < .01, ***P < .001

To clarify the expression of pseudouridine synthase in NSCLC, first, we analyzed the data derived from the TCGA to screen out the differentially expressed gene PUS7 in NSCLC from the pseudouridine synthase family (Fig. 1a). As is shown in the picture, the expression levels of PUS7 were significantly higher in lung squamous cell carcinomas and lung adenocarcinomas than in normal lung tissue (Fig. 1b). Then, four NSCLC cell lines were used to analyze the basic expression of PUS7. According to the results of RT-qPCR and Western blot, PUS7 expression was remarkably up-regulated in four NSCLC cell lines (H1299, A549, H226, and H460) compared with normal bronchial epithelial cells, BEAS-2B (Fig. 1c and d). Therefore, we selected A549 cells and H1299 cells, which were transfected with siRNA-PUS7 and pcDNA3.1-PUS7 for knockdown (KD) and overexpression (OE), respectively (Fig. 1e).

### PUS7 promotes NSCLC cells proliferation, migration, and invasion

**Fig. 2:**
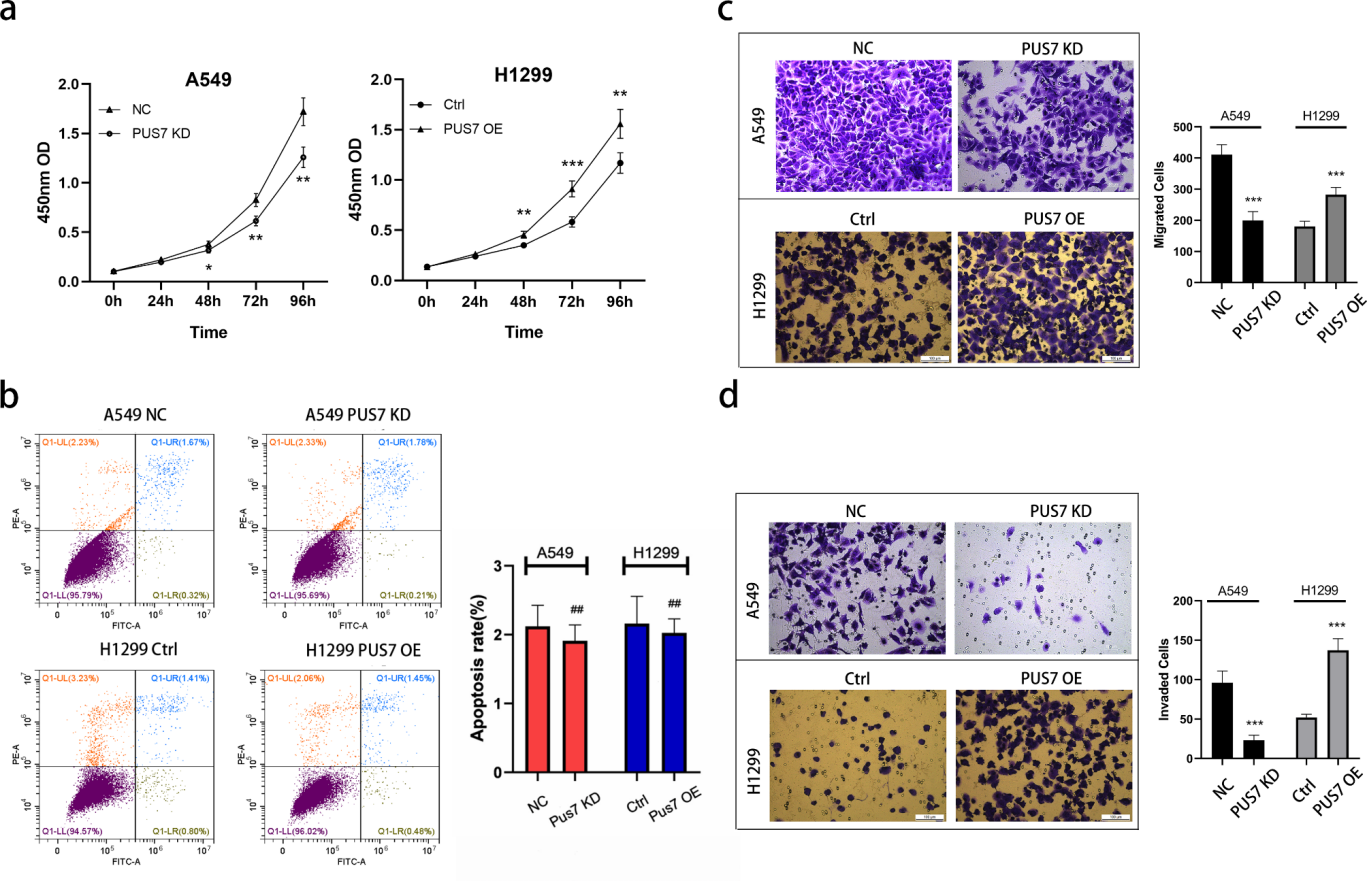
PUS7 regulates the proliferation, migration, and invasion of NSCLC cell lines. **(a)** Cell Counting Kit-8 was used to detect the proliferation ability of NSCLC cells. **(b)** Flow cytometry was used to detect apoptosis in NSCLC cells (*P* = .397 and 0.632). **(c)** A migration assay was used to assess NSCLC cell migration. **(d)** An invasion assay was used to determine NSCLC cell invasion. All the data are shown as the mean ± s.d. ##*P* > .05, *P < .05, **P < .01, ***P < .001

At 48 h, 72 h, and 96 h after PUS7 knockdown, the proliferation of PUS7-KD A549 cells was remarkably slower than that of the control group, whereas PUS7 overexpression exhibited a contrasting trend in H1299 cells (Fig. 2a). Similarly, PUS7 KD remarkably weakens migration ability and invasion ability in 549 cells, while PUS7 OE can promote migration and invasion of H1299 cells (Fig. 2c and d). As for the apoptosis of tumor cells, the results of flow cytometry indicated that neither knockdown nor overexpression of PUS7 affected the apoptosis level of NSCLC cells (*P* all *> 0.05*, Fig. 2b).

### High expression of PUS7 in tumor tissue suggests poor prognosis in NSCLC patients

**Fig. 3:**
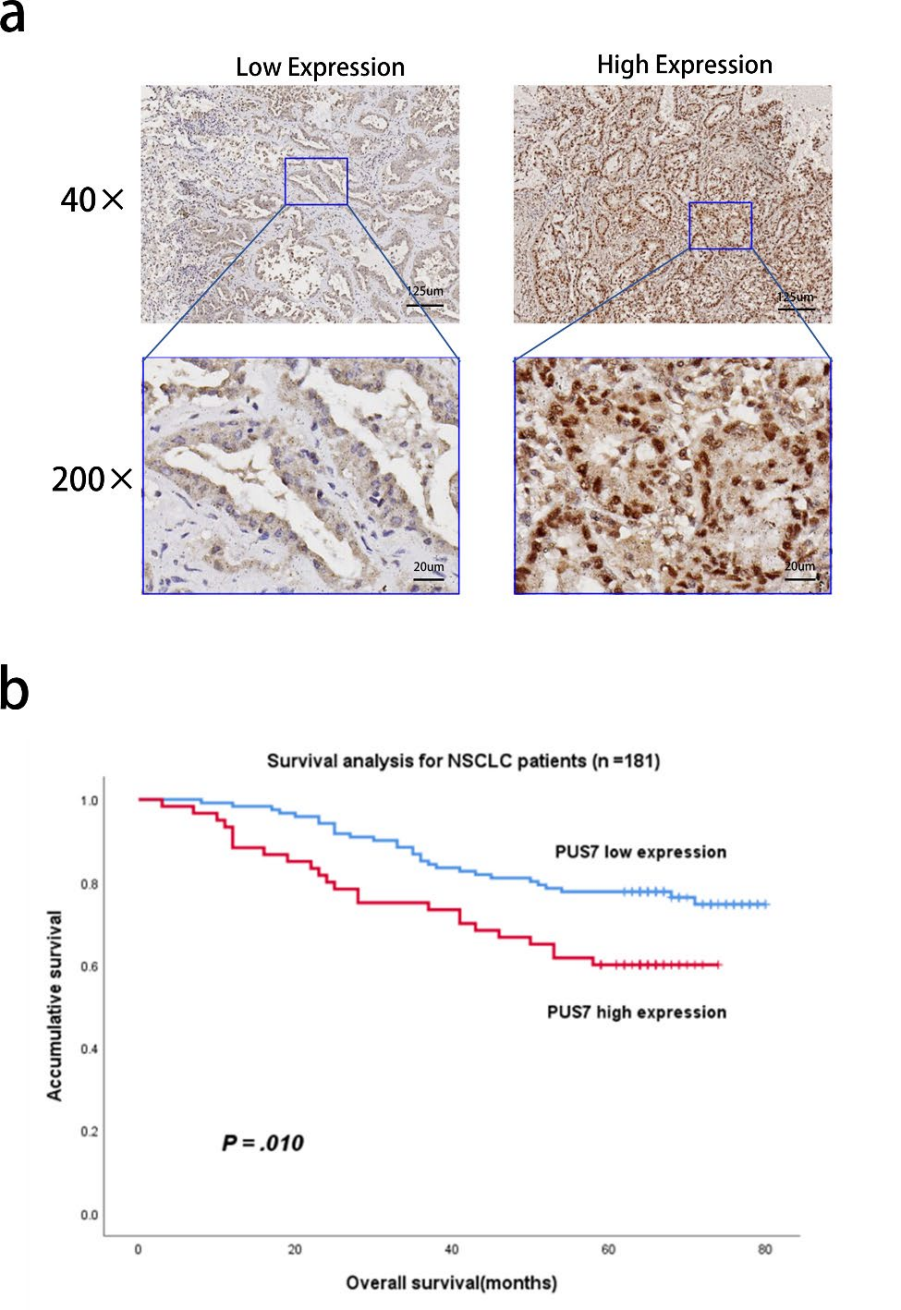
The expression of PUS7 in tumor tissue correlates with the prognosis of patients with non-small cell lung cancer. **(a)** Immunohistochemical staining was used to assess the expression of PUS7 in NSCLC tissues (40× and 200×). **(b)** Kaplan-Meier survival analysis was used to explore the association of PUS7 with the prognosis of NSCLC patients

After bioinformatics analysis and cell experiments, clinical data analysis was performed. We obtained pathological specimens from NSCLC patients in the First Affiliated Hospital, Sun Yat-sen University, and then performed immunohistochemical staining to evaluate the correlation between PUS7 and patient prognosis based on the expression of PUS7 in tumor tissues. Typical pictures of immunohistochemical staining are listed in Fig. 3a, and the two IHC-stained slides were scored 1 and 12, respectively, according to our pathological scoring criteria. X-tile software defined the optimal cutoff value, 8, and when the immunohistochemical scores were divided into low expression group (0–8) and high expression group (9–12), there was an obvious difference in the overall survival (OS) of NSCLC patients between the two groups (Supplement figure S1). Kaplan-Meier analysis data showed that the prognosis of patients in the PUS7 high expression group was distinctly worse (*P* = .010, Fig. 3b).

Then, the chi-square test was employed to explore the relation between PUS7 expression and the clinical characteristics of the patients, showing that the expression of PUS7 had nothing to do with patient gender, age, pathological type, differentiation, adjuvant chemotherapy, and clinical stage (*P* all > 0.05, Table 1).

**Table 1:** Correlation between the PUS7 expression and clinicopathological characteristics in human NSCLC tissues

	Variables	Cases	PUS7		*P*
			Low expression	High expression	
Gender	Male	106	66	40	0.119
	Female	75	55	20	
Age (years)	≤ 60	94	62	32	0.791
	> 60	87	59	28	
Pathological type	Non-adenocarcinoma	27	14	13	0.073
	Adenocarcinoma	154	107	47	
Differentiation	Moderate and poor differentiation	125	86	39	0.405
	High differentiation	56	35	21	
Clinical stage	Stage IB-IIB	137	94	43	0.486
	Stage IIIA	46	29	17	
Adjuvant chemotherapy	Received	107	67	40	0.146
	Not received	74	54	20	

Next, Cox regression analysis on clinical data was performed to evaluate the prognostic significance of PUS7 expression in NSCLC tissue. Univariate analysis showed that gender, PUS7 expression, tumor pathological type, adjuvant chemotherapy, and clinical stage were prognostic factors (*P* all < 0.05, Table 2). Further multivariate analysis data also confirmed that gender, clinical stage, adjuvant chemotherapy, and PUS7 expression were independent prognostic risk factors for NSCLC patients after surgery (*P* all < 0.05, Table 2).

**Table 2:** Univariate and multivariate analysis of the factors associated with overall survival in NSCLC patients

Variables	*P*	Hazard ratio	95% confidence interval
Univariate analysis			
Age (< 60 vs. ≥ 60)	0.609		
Differentiation (I-II vs. III)	0.095		
Gender (Female vs. Male)	0.007	0.429	0.233–0.79
PUS7 (high vs. low)	0.012	2.007	1.164–3.461
Pathological type (Non-adenocarcinoma vs. Adenocarcinoma)	0.020	0.466	0.245–0.888
Clinical stage (IIIA vs. IB-IIB)	< 0.001	7.348	4.193–12.875
Adjuvant chemotherapy (Received vs. Not received)	0.033	0.555	0.324–0.953
Multivariate analysis			
Gender (Female vs. Male)	0.014	0.456	0.244–0.853
PUS7 (high vs. low)	0.007	2.148	1.237–3.730
Clinical stage (IIIA vs. IB-IIB)	< 0.001	10. 381	5.779–18.647
Adjuvant chemotherapy (Received vs. Not received)	< 0.001	0.296	0.166–0.529

## Discussion

The widespread pseudouridine modification of RNA in human cells stabilizes RNA conformation and disrupts various RNA-associated proteins, implying that pseudouridylation may widely influence RNA synthesis and breakdown, even gene expression [16, 26]. Recent studies have shown that the median level of PUS7 mediated pseudouridine modification is 10% [27]. Aberrant expression of pseudouridine synthase can result in dysregulation of the process of RNA pseudouridylation, suggesting its potential oncogenic significance [8, 9, 28].

Currently, there is no study of PUS7 in NSCLC. Bioinformatics analysis showed that PUS7 was differentially expressed between NSCLC tissues and adjacent tissues. Through cell experiments, compared with normal cell lines, we verified that PUS7 is highly expressed in NSCLC cell lines, which can boost the proliferation, migration, and invasion of NSCLC cells. However, the expression level of PUS7 did not affect the apoptosis of NSCLC cells. It is similar to the findings of PUS7 in other malignancies, which have been reported to affect glioblastoma growth and tumorigenesis by regulating TYK2 translation through tRNA-dependent pseudouridylation [22]. In addition, studies have shown that PUS7 can influence colon cancer cell metastasis by regulating LASP1, but does not affect tumor cell proliferation [24]. However, another study suggests that PUS7 also can activate the Wnt/β-catenin pathway by acting on Sirtuin 1 [29] and PI3K/AKT/mTOR signaling pathway [30], indicating that PUS7 may simultaneously act on multiple downstream targets and promote colorectal cancer progression. The above studies suggest that PUS7 plays different roles in different malignant tumors. Therefore, targeted inhibition of PUS7-mediated pseudouridine modification may be a promising therapeutic approach, but the lack of specific inhibitors of pseudouridine synthase has retarded the research on related targeted therapies. In general, there are still few studies on pseudouridine synthase in tumors, and we need to conduct additional research to elucidate its specific carcinogenic mechanism.

Recently, some studies have reported that PUS7 can be considered a possible biomarker for ovarian cancer diagnosis [23] and as a prognostic predictor for glioma [21]. Our study shows that PUS7 expression in tumor tissue is an independent prognostic factor for NSCLC patients, and the OS of NSCLC patients with high expression of PUS7 in tumor tissue is significantly shorter. It means that PUS7 is a potential biomolecule to assess the prognosis of NSCLC patients after surgery, and provides clues for clinicians to formulate treatment plans. In addition, Jin z et al. found that multiple PUS family genes, such as DKC1, PUS1, and PUS7, are upregulated in hepatocellular carcinoma and are associated with poor prognosis [11, 31]. There are also studies that confirm that PUS7 is involved in the occurrence of hematologic tumorigenesis. For instance, PUS7-mediated ψ activates certain tRNAs to control protein synthesis and influence stem cell differentiation, and dysregulation of this process may lead to myelodysplastic syndrome (MDS) worsening into acute myeloid leukemia (AML) [32, 33]. Based on the above research, we raise a question, are the PUS genes widely involved in tumor occurrence and progression? It remains a mystery due to limit research. Obviously, it is worthwile to pay more attention on this field.

Of course, there are still some shortcomings in this study. First, although our study confirmed the high expression and role of PUS7 in NSCLC, we still do not know the relevant targets of PUS7 and whether PUS7 acts on downstream molecules in an RNA pseudouridylation-dependent manner. In addition, we only investigated the significance of PUS7 in NSCLC cytology without animal experiments. Finally, the number of patients in our study is relatively insufficient, and it is only a single-center retrospective study, which still needs to be further confirmed by a multi-case and multi-center prospective study.

## Conclusion

PUS7 can promote the progression of NSCLC, and the higher expression of PUS7 in tumor tissue indicates poor prognosis of patients, which may be a biomolecule that affects prognosis.

## Electronic supplementary material

Below is the link to the electronic supplementary material.

Additional File 1: The results of X-tile analysis demonstrate the optimal cutoff value between low and high expression groups

## Data Availability

The data used to support the findings of this study are included within the article. The data and materials in the current study are available from the corresponding author on reasonable request.
