# Supplementary figures and images for: Higher expression of pseudouridine synthase 7 promotes non-small cell lung cancer progression and suggests a poor prognosis
Source: J Cardiothorac Surg. 2023 Jul 7;18:222. doi: 10.1186/s13019-023-02332-z (PMC10329395; doi:10.1186/s13019-023-02332-z)

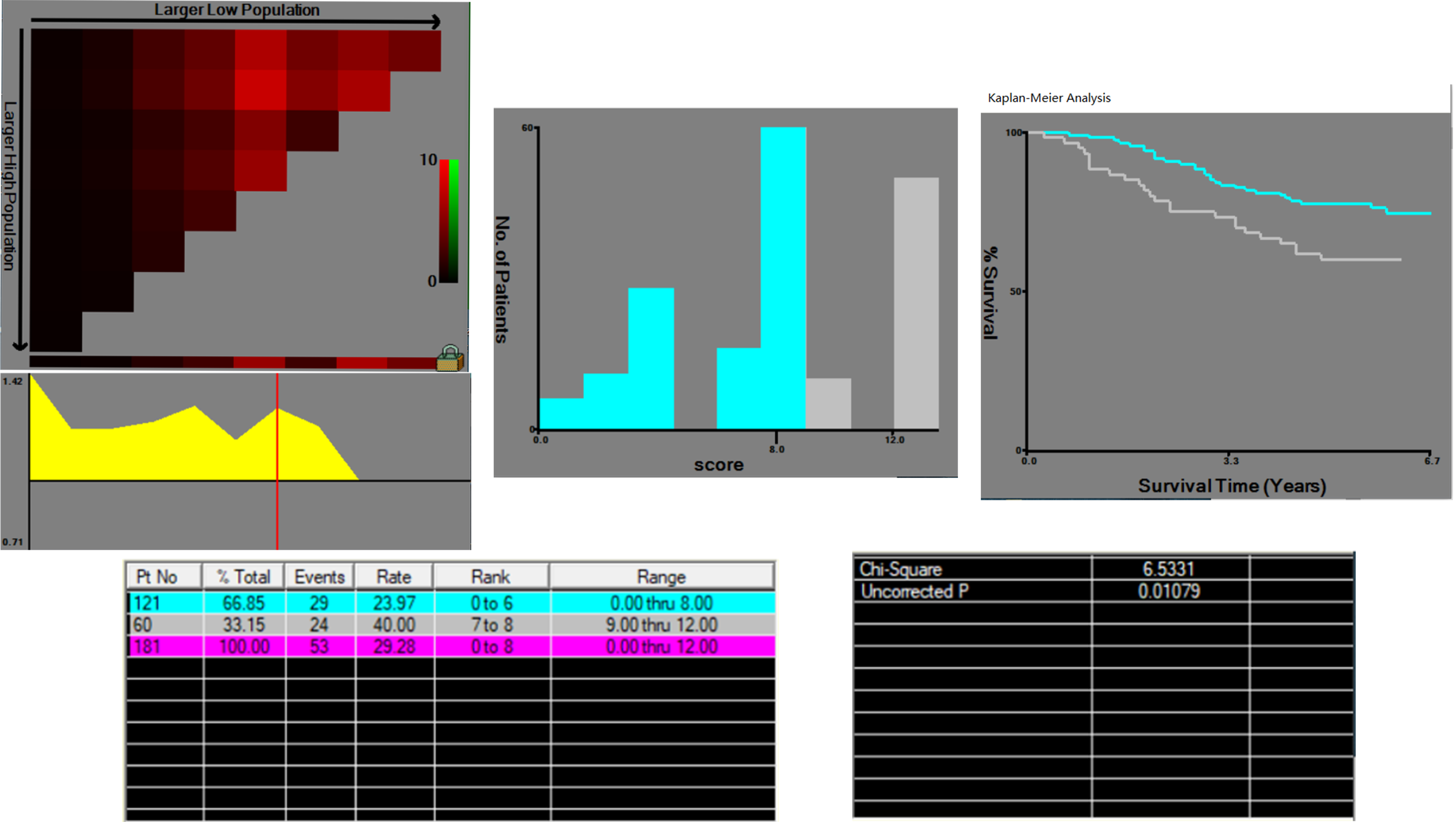

Supplement: Supplementary file 1 — Additional File 1: The results of X-tile analysis demonstrate the optimal cutoff value between low and high expression groups [file 13019_2023_2332_MOESM1_ESM.tif]
